# The effect of professional portfolio learning on nursing students’ professional self-concepts in geriatric adult internship: a- quasi-experimental study

**DOI:** 10.1186/s12909-023-04097-4

**Published:** 2023-02-15

**Authors:** Samira Mohajer, Tang Li Yoong, Chong Mei Chan, Mahmoud Danaee, Seyyed Reza Mazlum, Nasser Bagheri

**Affiliations:** 1 Nursing and Midwifery Care Research Center, Mashhad University of Medical Sciences, Mashhad, Iran; 2 Department of Nursing Science, Faculty of Medicine, University of Malaya, Kuala Lumpur, Malaysia; 3 Department of Social and Preventive Medicine, Faculty of Medicine, University of Malaya, Kuala Lumpur, Malaysia; 4 Visual and Decision Analytics (VIDEA) lab, Health Research Institute, University of Canberra, The National Centre for Epidemiology and Population Health, College of Health and Medicine, The Australian National University, Canberra, Australia

**Keywords:** Professional self-concept, Professional portfolio, Blended learning, Nursing students, Geriatric-adult internship practice

## Abstract

**Background:**

Professional self-concept is one of the important outcomes of nursing professionalism. There is a lack of adequately planned curriculum may limit nursing students’ practical knowledge, skills and professional self-concept in providing comprehensive geriatric-adult care and promoting nursing professionalism. Professional portfolio learning strategy has allowed nursing students to continue professional development and enhance nursing professionalism in professional clinical practice. However, there is little empirical evidence in nursing education to support the use of professional portfolios in blended learning modality among internship nursing students. Therefore, this study aims to examine the effect of the blended professional portfolio learning on professional self-concept among undergraduate nursing students during Geriatric-Adult internship.

**Methods:**

A quasi-experimental study two-group pre-test post-test design. A total of 153 eligible senior undergraduate students completed the study (76 in the intervention group and 77 in the control group). They were recruited from two Bachelor of Sciences in Nursing (BSN) cohorts from nursing schools at Mashhad University of Medical Sciences (MUMS), in Iran, in January 2020. Randomization was undertaken at the level of school via a simple lottery method. The intervention group received the professional portfolio learning program as a holistic blended learning modality, though the control group received conventional learning during professional clinical practice. A demographic questionnaire and the Nurse Professional Self-concept questionnaire were used for data collection.

**Results:**

The findings imply the effectiveness of the blended PPL program. Results of Generalized Estimating Equation (GEE) analysis was indicated significantly improved professional self-concept development and its dimensions (self-esteem, caring, staff relation, communication, knowledge, leadership) with high effect size. The results of the between-group comparison for professional self-concept and its dimensions at different time points (pre, post and follow up test) showed a significant difference between groups at post-test and follow up test (p < 0.05),while at pre-test there was no important dissimilarity between two groups (p > 0.05).The results of within-group comparison for both control and intervention showed that there were significant differences in professional self-concept and for all its dimensions across the time from pre-test to post-test and follow-up (p < 0.05), and also from post-test to follow-up it was significant (p < 0.05) for both groups.

**Conclusion:**

This professional portfolio learning program demonstrates as an innovative and holistic blended teaching-learning approach to improve professional self-concept during professional clinical practice among undergraduate nursing students. It appears that the use of a blended designed of professional portfolio can promote a link between theory and the advancement of geriatric adult nursing internship practice. The data obtained from the present study can be useful for nursing education to evaluate and redesign a curriculum for development of nursing professionalism as a quality improvement process and groundwork to develop new models of teaching-learning and assessment.

## Background

Nursing professionalism envelops a set of values that are basic to raising the quality of patient care whereas making strides the educational learning strategies, benchmarks, and judgments that direct nursing practice each day [1]. The description of professionalism helps to find the ways to the development of professional and professional roles to promote nurses’ professional self-concept [2] and can increase professional motivation and improve the provision of holistic care to different physical ,mental and social health needs of geriatric and adult patients [3, 4]. Professional Self-Concept (PSC) as a multidimensional concept, refers to an individual’s perception of self as a professional person, affecting his/her way of thinking, role evolution, professional behavior and performance, and self-confidence [5]. According to Cowin (2008), developer of self-concept theory of Cowin’s model, strong self-concept contributes to the positive development of professional self [6]. Besides, a low PSC adds the rate of quitting the job and care errors and decreases the quality and quantity of nursing care [7]. Many studies in Iran and other countries evaluated the level of professional self-concept among nurses and nursing students and their results respectively have shown a low to moderate level of professional self-concept [8–11].

Over the past two decades, the studying clinical environment in nursing practice education has received a bigger interest [12, 13, 14, 15]. Clinical internship is a very important part of nursing education; it provides students with opportunities to practice what they have learned in the classroom, helping them to enhance their knowledge and skills to respond to everyday practice situations and to internalize nursing students’ positive recognition of nursing, professional attitudes, and develop professional self-concept [16]. Results of recent study by Mohajer et al. in 2022 reported a moderate level of total PSC and the lowest dimensions in self-esteem among nursing students in Iran during internship nursing practice [17]. Therefore, a professional nurse is required to exhibit a PSC that has been developed during the professional education program [18], which can boost nursing professionalism [17]. Currently, in the nursing curriculum of Iran and other aging countries there is a lack of systematically planned clinical teaching approaches that focus on the holistic care of both geriatrics and adults patients with different health problems. Such a lack of adequately planned curriculum may limit nursing students’ practical knowledge, skills and professional self-concept in providing comprehensive geriatric-adult care and promoting nursing professionalism [19]. In nursing education, Professional Portfolio learning (PPL) is one educational approach for developing nursing professionalism to help students link theory and practice [20]. A professional portfolio (PP) is a systematic collection of student work that represents student activities, accomplishments, and achievements over a specific period of time in one or more areas of the curriculum. The educational value of PPL through student-centered learning can promote reflective and deep learning. According to the literature reviews, Professional portfolio learning (PPL) may assist nursing students to improve in knowledge, skills, attitudes, self-learning, study satisfaction, critical thinking, problem-solving skills, and professional competency [19–26]. Although previous studies reported the PPL approach was associated with positive outcomes, due to inadequate studies on the effectiveness of this method, it’s not systematically implemented and structured in Iranian nursing education [21]. On the other hand, nowadays, online learning is one essential educational strategy but is not an effective alternative for nursing education. With this technology, portfolios have transitioned from paper form to electronic form [26]. Meanwhile, nursing cannot be taught exclusively online and achieve satisfactory educational outcomes [27]. Accordingly, Professional portfolio learning (PPL) in blended learning modality is a holistic learning program to stimulate and monitor students’ professional development and can promote their ability to become lifelong learners [20]. This holistic program not only accepts the intention of mixing online learning and face-to-face learning but also emphasizes the integration of many teaching models, ultimately constituting a combination of learning resources, learning styles, and learning environments [28].

Given the lack of empirical evidence in the PPL in Geriatric-Adult internship program with blended teaching-learning strategy and concerning the urgent attention to improving PSC among Iranian nursing students, to the best of our knowledge, this has not been studied in the nursing education of Iran. Therefore, development of blended PPL can engage professional development activities such case studies, reflective practice, group discussions, and simulation debates seminar during the blended program sessions in professional clinical practice and so can lead to promoting nursing professionalism in nursing students and improving quality of care in geriatric/adult patients. The evidence on the effectiveness of existing portfolio approaches also remains limited, which in turn restricts the ability of nursing educators to incorporate such approaches into the curriculum. Therefore, this study aims to examine the effect of the blended professional portfolio learning on professional self-concept among undergraduate nursing students during Geriatric-Adult internship.

## Methods

### Study design

This quasi-experimental study was conducted in 2020 using a two-group, pre-test post-test design.

### Setting and participants

**Fig. 1:**
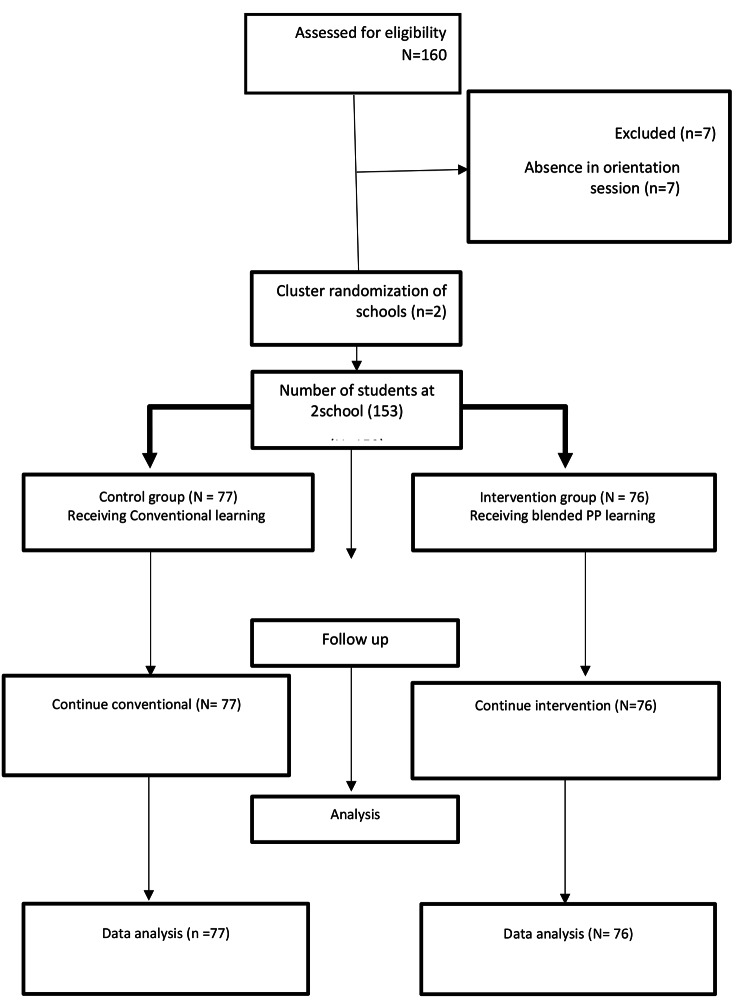
Flow chart of data collection process

The senior undergraduate nursing students were recruited from two Bachelor of Sciences in Nursing (BSN) cohorts from Mashhad School of Nursing & Midwifery at Mashhad University of Medical Sciences (MUMS), in Iran, in January 2020, in the North-East Province of Iran, who finished the academic program and take their first professional clinical practice in Clinical Geriatric-Adult Nursing (CGAN) Course. Probability-sampling methods of cluster random sampling were used to recruit participants. Cluster random sampling involves selecting groups rather than selecting individuals. Randomization via a simple Lottery method was undertaken at the level of school rather than with individual students to avoid contamination from the intervention group to the control group. Therefore, two schools randomly allocated to intervention or control groups. The calculation of sample size was based on the effect sizes of similar studies by [22] & [8] the minimum sample size was calculated from the formula of effect size and the results were 70 in each group. Taking into consideration an attrition rate of 20%, the adjusted total sample size was 160. Four students in the intervention and three in the control groups were absent at the time of the baseline data collection, and all the153 students remained (76 in the intervention group and 77 in the control group) and completed the study. There were no any drop out across the study. Therefore the response rate was 100% (Fig. 1).

The Inclusion criteria were signing up for this course and having no previous experience of PPL. Had a smartphone with internet access in their residence and be able to use it effectively (intervention group) and should attend professional internship clinical practice for sixteen weeks during the program and fulfill all of the activities. The exclusion criteria failed to accomplish the requirement of the PPL program, and students were excepted if they partially responded to study questionnaires or had more than one absence from the course sessions. The eligible senior nursing students in intervention and control groups had 16 weeks of professional clinical practice during the intervention period. All students provided written informed consent to participate. All methods were carried out in accordance with relevant guidelines and regulations or declaration of Helsinki.

### Research instruments and data collection

Firstly, the qualified nursing students were recognized from the authoritative record and they were inquired to take an interest in this to consider through the welcome letter (Fig. 1). At that point, data was accumulated employing a survey comprising of two segments. The first segment collected respondents’ demographic data “gender, age, marital status, clinical experience, nursing interest and, Grade Point Average (GPA)”. The second section consists of two-part, first the Nurse Professional Self-concept (NPSC) developed by Cowin, (2008). The tool is a 36-item questionnaire; it addresses the following six dimensions among nurses: self-esteem (6 questions), knowledge (6 questions), caring (6 questions), leadership (6 questions), staff relations (6 questions), and communication (6 questions). Each subscales consisting of six items in six-point Likert scale form (1 = “I totally disagree” ,2 = disagree 3 = relatively disagree 4 = relatively agree 5 = agree and 6 = “I totally agree”). All items are expressed positively and scored based on a six-point Likert scale, and the score range of the tools is 36–216 (6–36 range of score for each dimension) and a higher score shows an increased level of PSC. The reliability and validity of the NPSC have been verified by several studies conducted abroad and Cronbach’s alpha was reported between 0.82 and 0.92 in the study conducted by Cowin in 2008, [6]. Previously in Iran, the validity and reliability of the Persian version of this questionnaire were approved in different studies [9, 29, 30]. The Persian version has a Spearman-Brown correlation coefficient of 0.84 and Cronbach’s alpha of 0.97, which indicate its reliability and validity. In the currents study the validity of the Persian version of the instruments was reassessed and approved using experts’ views of a panel of ten professors in nursing education, and also three nursing students, who were not in the actual study, verified the clarity of the questionnaire and its translated versions. The researcher asked them to read the instrument and provide the evaluation of the content, whether or not it has reflected the concept that the researcher planned to measure, before the study. Besides, the internal consistency of the tools was re-assessed to evaluate its reliability in the present study, twenty nursing students accidentally picked from the study setting, accomplished the PSC. Cronbach’s alpha was obtained to be 0.949 and the coefficient Correlation between the dimensions of the questionnaire was variable from 0.763 to 0.867. Both groups were asked for completing the questionnaires before starting the intervention (T0,). The second measurement (posttest) was performed on twelve-week (T1,). The third measurement was conducted in the sixteen weeks as a follow-up measurement (T2).

### Intervention and control group

The professional portfolio learning (PPL) program in the blended learning modality was a holistic structured intervention and consists of four professional development modules; portfolio learning, case study, reflective witting, and simulation seminar. Therefore, this holistic program involved a systematic combination of face-to-face and online teaching-learning approach mediated interactions between students, facilitators, researcher, and learning resources. In this light, blended learning offers the chance to perform asynchronously, allowing novel kinds of communication and teamwork. Also, the instructor has the chance to track and accompany the student learning process during the way and in the ending, to assess it based on various basics as the contribution, reflections, learning products, and feedback [31].

The intervention program was implemented in three phases according to some theories and models as its theoretical framework. Face-to-face activities guided through principles of Bandura’s Social Learning Theory (SLT). The learning process takes put through observation, imitation, and modeling that’s arranged by four standards process to be attention, retention, motor reproduction, and inspiration process [32]. Moreover, to back the blended approach of the PPL was in turn supported by a multi-theories model of adult learning, proposed by Taylor and Hamdy [33] and built upon the platform system that bolstered the complete internship nursing educational programs. Inside this theory, a five-stage demonstration of instruction was performed: Dissonance, refinement, organization, feedback, and consolidation.

Also during the clinical practice, the case study activity was implemented based on Continuing Professional Development (CPD) Model, and reflective writing activity was performed based on Holistic Reflection Model (HRM) by Bass et al. (2017) and to support reflective practice [34] and learning from each other while working together and develop their professional portfolio. Phase I includes pre-intervention to prepare nursing students and their facilitators to undertake the PPL program initiative. Phase II provides training and implementation of the PPL. The last phase was the follow-up part.

### Phase 1: pre-intervention

At the beginning of the intervention in the orientation session, the overall and the daily syllabuses of the Geriatric-Adult Nursing Course were delivered to students in both groups, then researcher in the intervention group created the online Telegram groups of each eight students in a group named Portfolio Learning Group (PLG). Ten clinical facilitators who directed and guided the students’ clinical practice were welcomed to go to a one-day workshop preparing session. The preparing was expecting to clarify the PPL and disseminate rules on how to convey it in clinical practice to assure their tasks and parts during the PPL program.

### Phase 2: training and implementation of PPL intervention

The professional portfolio learning (PPL) program has consisted of two-part preclinical and clinical. Part one (Preclinical) was teaching; the four-day workshop was run for nursing students teaching modules activities of PPL at week 1 before entering the clinical practice. This four-day workshop consists of four blended modules; portfolio learning, case study, reflective writing, and simulation seminar oriented them to PPL concepts including definition, benefits, and techniques of portfolio learning. Also, reviewing the definition and importance of PSC promotion and strategies provided. They were also needed to simulate and practice the modules that would be completed during their sixteen weeks of professional clinical practice. The workshops were held twice a day for a duration of four-day from 1 to 4 February 2020. The briefing notes and teaching contents remained available in the online groups for students’ reference.

Part two (Clinical) was implementation the intervention; Implementing modules activity during the professional clinical practice for Geriatric-Adult Nursing Course from week 2-week 16 and nursing students practice activities and developed their PP under face to face supervision and guidance from the facilitators. Besides, they received online teaching, activity assessment, and written feedback from the researcher. Clinical facilitators were assigned to oversee the PPL for a PLG during the course, in addition to fulfilling their usual student advising responsibilities. Case studies had chances to lead the student from a holistic perspective of the patients’ needs of nursing, they could see clear benefits and were satisfied with the case study analyzing [17]. Creating and maintaining a PP can also enable nursing students to identify their strengths and learning needs, and to improve a learning plan to address these requirements [35].

Based on the clinical program schedule, students were responsible for the care of their assigned both geriatric and adult patients and implemented a case study in their group based on the principles of the CPD model that comprised of six processes consists of; briefing, identifying learning needs, reflection, developing a plan and carry out, record and review, analyses, and evaluation to develop the professional portfolio in practice. Each student in PLG is expected to do at least two care plan activities for two patients (one geriatric and one adult) per week. Also, nursing students in PLG performed two reflective writing based on steps of the Holistic Reflective Model (HRM) [34]. In week six nursing students in PLG implemented a clinical seminar and simulated two patient scenarios (geriatric-adult). All activities were guided by the facilitator, then share in the online group at the end of the each weeks to comprehensive formative assessment and received written feedback from the researcher.

Thus, five sessions of PPL (2 case studies, 2 reflective writing & 1 simulation seminar) were performed by the students during week2-week6. The students also implemented four individual sessions of PLP during weeks 7–10 of clinical practice. On the last day of each week, professional activities of PPL were posted to the discussion online group were reviewed by the researcher after the students finished each section, and relevant feedback and responses were posted for the group to see. Week 11 researcher asked students to improve their PP constantly and they reviewed their activities regularly with a serious consideration of the researcher feedback and gained submitted their revised PP to the researcher (summative assessment). In week 12 students worked on the posttest assessment section.

### Phase 3: follow up

**Fig. 2:**
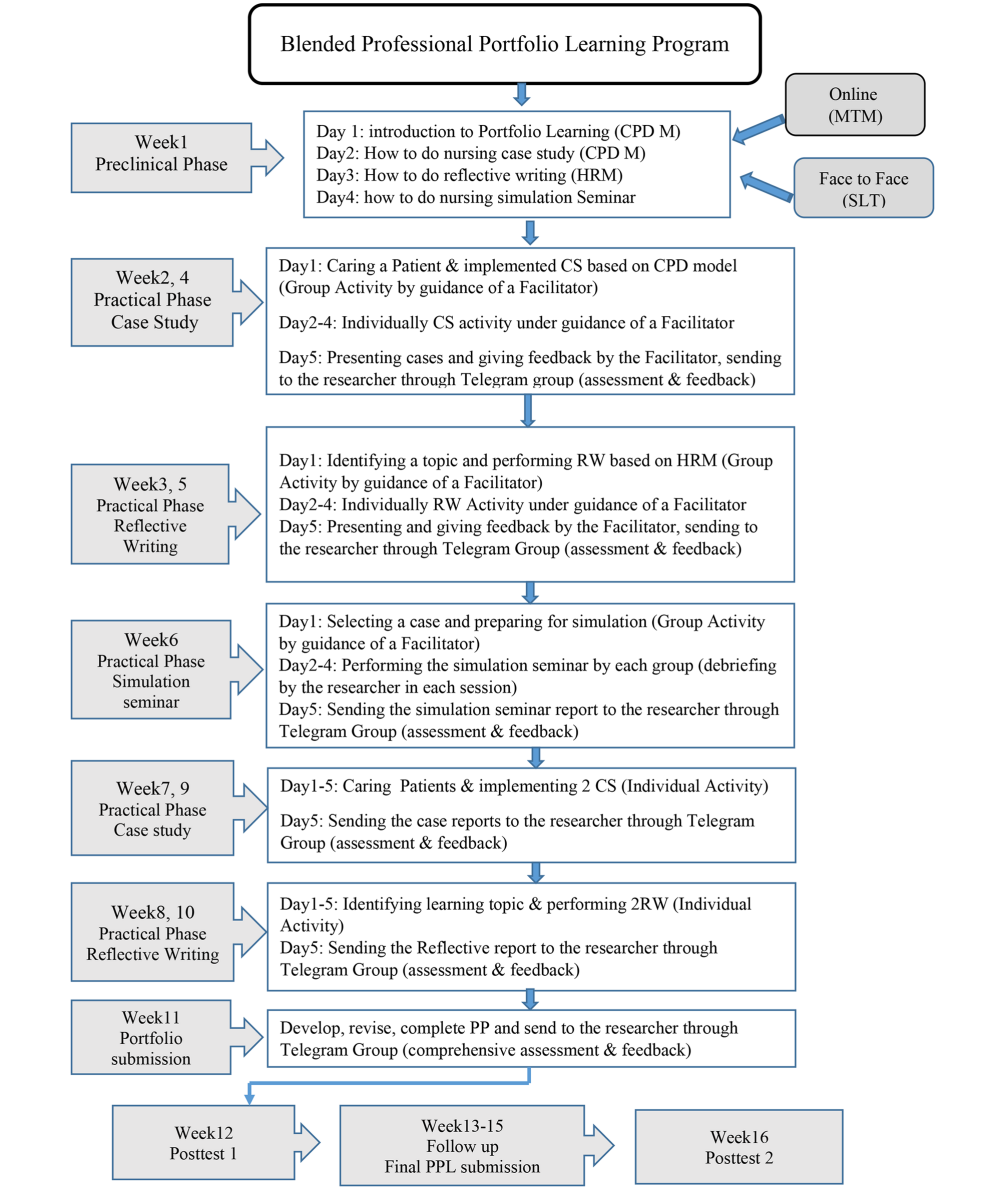
The Phses of Blended Professional Portfolio Learning Program

From week 13–15 as a follow-up part, students independently continue to utilize case studies and reflective practice writing as well as other features to compile their PP. Students to aim improve PSC were needed to reveal and share their interactions with patients and family, to estimate (and perhaps revise) their nursing care plan, and to assess learning results in professional practice. The researcher invited the PLG to share their complete PP with the online group for a comprehensive assessment. Then, students completed all professional development activities of the PPL program, revised, and compiled the PP items. Finally, students developed their professional portfolios. Figure 2 shows the protocol of how the blended PPL program runs in the professional clinical practice of Clinical Geriatric-Adult Nursing (CGAN) course in medical-surgical wards, (Fig. 2).

Students in the control group undertook the clinical practice using the conventional learning system and were responsible to complete and submit their logbooks. The control group participants were given online access to the content of the PPL program upon completion of the study.

### Data analysis

The data were analyzed utilizing the SPSS software program Form 25.0. Descriptive statistics, Chi-Square test, and Independent T-test were conducted to compare both groups for “participants’ characteristics” and study outcomes at baseline. The Generalized Estimating Equations (GEE) was utilized to assess the impact of the PPL program over time, between the interventional and control groups, and within the interaction between the time and group. Due to the significant differences in the PSC and its dimensions at pre-test and age for intervention and control groups, this variable was considered as a covariate in the analysis, therefore a GEE analysis was applied to assess whether there were groups and times differences in respondent’s PSC and its dimensions.

## Results

### Participants characteristics

The number of qualified students was 153 and none of them were excluded from the study. There were no any drop out across the study. Therefore the response rate was 100%. The results of the Chi-Square test and Independent t-test (Table 1) showed no important dissimilarity between both groups in the baseline scores for any of the study results, except in age, which was significantly different between intervention and control groups, therefore age was considered as a covariate for all analysis. Furthermore, the results of comparing two groups at baseline indicated that there were no considerable dissimilarities between the two groups for total PSC and its dimensions. Table 2 shows the descriptive statistic (mean and CI95%) of PSC and its dimensions for both groups at three times.

**Table 1:** Comparison of the participants’ characteristics between the experimental and control groups at baseline for intention-to-treat (ITT) analysis

Variable	Level	Control group		Intervention group		χ^2^ /t	P-value
		n	%	n	%		
Gender	Male	26	33.8	26	34.2	0.003	0. 954
	Female	51	66.2	50	65.8		
Marital status	Single	51	66.2	58	76.3	1.89	0.17
	Married	26	33.8	18	23.7		
Clinical experience	Yes	71	92.2	66	86.8	1.17	0.28
	No	6	7.8	10	13.2		
Nursing interest	High	64	89.5	68	83.1	1.3	0.25
	Low	13	16.9	8	10.5		
age	Mean ± SD	22.32 ± 0.98		22.8312 ± 1.71			< 0.001
GPA	Mean ± SD	17.2373 ± 1.1156		16.9725 ± 0.92501		-1.597	0.112

*χ2 = Chi-square; P-value = level of statistical significance (p < 0.05)*

**Table 2:** Professional self-concept and its dimensions mean scores within and between

PSC	Time	Intervention Group		Control Group	
		**Mean**	**95% CI** **(LL, UL)**	**Mean**	**95% CI** **(LL, UL)**
Self-esteem	Pre-Test	25.6	22.8, 28.5	25.3	22.63, 28.09
	Post-Test	30.4	27.61, 33.2	25.5	22.85, 28.30
	Follow-up	31.5	28.81,34.36	25.8	23.09, 28.53
	Pre-Test	27.2	23.5,30.8	26.9	23.4, 30.5
Caring	Post-Test	30.4	26.8,34.1	27.2	23.6, 30.7
	Follow-up	31.5	27.9, 35,2	27.4	23.6, 31
	Pre-Test	26.6	20.8,32.4	27	21.3,32.6
Staff relation	Post-Test	29.9	24.,35.7	27.4	21.7, 33
	Follow-up	31.9	26.1, 37.7	27.4	21.7, 33.1
	Pre-Test	27.9	24.8, 31.9	27.5	24.5, 30
Communication	Post-Test	30.1	27.3, 33.3	28.1	25,31.1
	Follow-up	32.1	29, 35.2	28.4	25.4,31.4
	Pre-Test	27.3	23.2, 31.4	27.6	23.6,31.7
Knowledge	Post-Test	30.6	26.4, 34.7	28	24.06, 32.08
	Follow-up	32.1	28.1, 36.2	28.3	24.3, 32.3
	Pre-Test	26.4	22, 30.9	26.3	21.9, 30.7
Leadership	Post-Test	29.4	24.9, 33.9	26.6	22.1,31
	Follow-up	31.1	26.5, 35.6	26.8	22.3, 31.2
	Pre-Test	26.49	22.0,30.90	26.36	21.94,30.78
Total Professional self -concept	Post-Test	29.48	24.97,33.99	26.61	22.19,31.02
	Follow-up	31.11	26.59,35.63	26.80	22.39,31.22

*Pre-test (1 week); Post-test (12th weeks); Follow-up (16th weeks), CI = Confidence Interval*

### The Effect of professional portfolio learning program on Professional self-concept (PSC) and its dimensions

Professional self-concepts(PSC) had significantly increased in the intervention group. Results of Generalized Estimating Equation (GEE) showed that there was a significant interaction between time and group on total PSC, self-esteem ,caring ,staff relation, communication, knowledge and leadership indicating that the score of PSC and its dimensions were significant across the time ( pre, post and follow up test) between two groups, therefore post hoc test using Bonferroni test was employed to compare the mean scores between and within groups. The results of the between-group comparison (Table 3) for PSC and its dimensions at different time points (pre, post and follow up test) showed a significant difference between groups at post-test and follow up test (p < 0.05) while at pre-test there was no important dissimilarity between two groups (p > 0.05).

**Table 3:** Results of generalized estimating equations (GEE) in Total Professional self-concept and its dimensions

Variable	Test	(I) Group	*(J)* Group	Mean Difference (I-J)	SE	P value	95%CI for Difference LB UB		effect size d
Total Prof self-concept	Pre-test	I	C	0.31	0.56	0.581	-0.79	1.41	5.3 (Large)
	Post-test	I	C	15.73	0.76	< 0.001	14.23	17.22	
	Follow up	I	C	25.8^*^	0.77	< 0.001	24.27	27.33	
	Pre-test	I	C	0.33	0.19	0.085	-0.04	0.77	3.1 (Large)
Self-esteem	Post-test	I	C	4.82	0.23	< 0.001	4.3	5.2	
	Follow up	I	C	5.77	0.24	< 0.001	5.3	6.2	
	Pre-test	I	C	0.22	0.15	0.158	− 0.08	− 0.52	1.5 (Large)
Caring	Post-test	I	C	3.28 ^*^	0.2	< 0.001	2.8	3.6	
	Follow up	I	C	4.13^*^	0.19	< 0.001	3.7	4.5	
	Pre-test	I	C	− 0.34	0.27	0.204	− 0.87	0.18	2.2 (Large)
Staff relation	Post-test	I	C	2.52 ^*^	0.28	< 0.001	1.97	3.08	
	Follow up	I	C	4.49 ^*^	0.31	< 0.001	3.87	5.1	
	Pre-test	I	C	0.35	0.19	0.068	− 0.02	0.73	2.2 (Large)
Communication	Post-test	I	C	2.05 ^*^	0.23	< 0.001	1.5	2.5	
	Follow up	I	C	3.66 ^*^	0.23	< 0.001	3.2	4.3	
	Pre-test	I	C	− 0.34	0.27	0.108	− 0.77	0.07	2.5 (Large)
Knowledge	Post-test	I	C	2.52 ^*^	0.25	< 0.001	2.01	3.03	
	Follow up	I	C	3.83 ^*^	0.21	< 0.001	3.4	4.2	
	Pre-test	I	C	0.13	0.17	0.447	− 0.20	0.47	2.4 (Moderate)
Leadership	Post-test	I	C	2.87 ^*^	0.24	< 0.001	2.3	3.3	
	Follow up	I	C	4.30 ^*^	0.25	< 0.001	3.8	4.8	

* The mean difference is significant at the 0.05 level Adjustment for multiple comparisons: Bonferroni

**Fig. 3:**
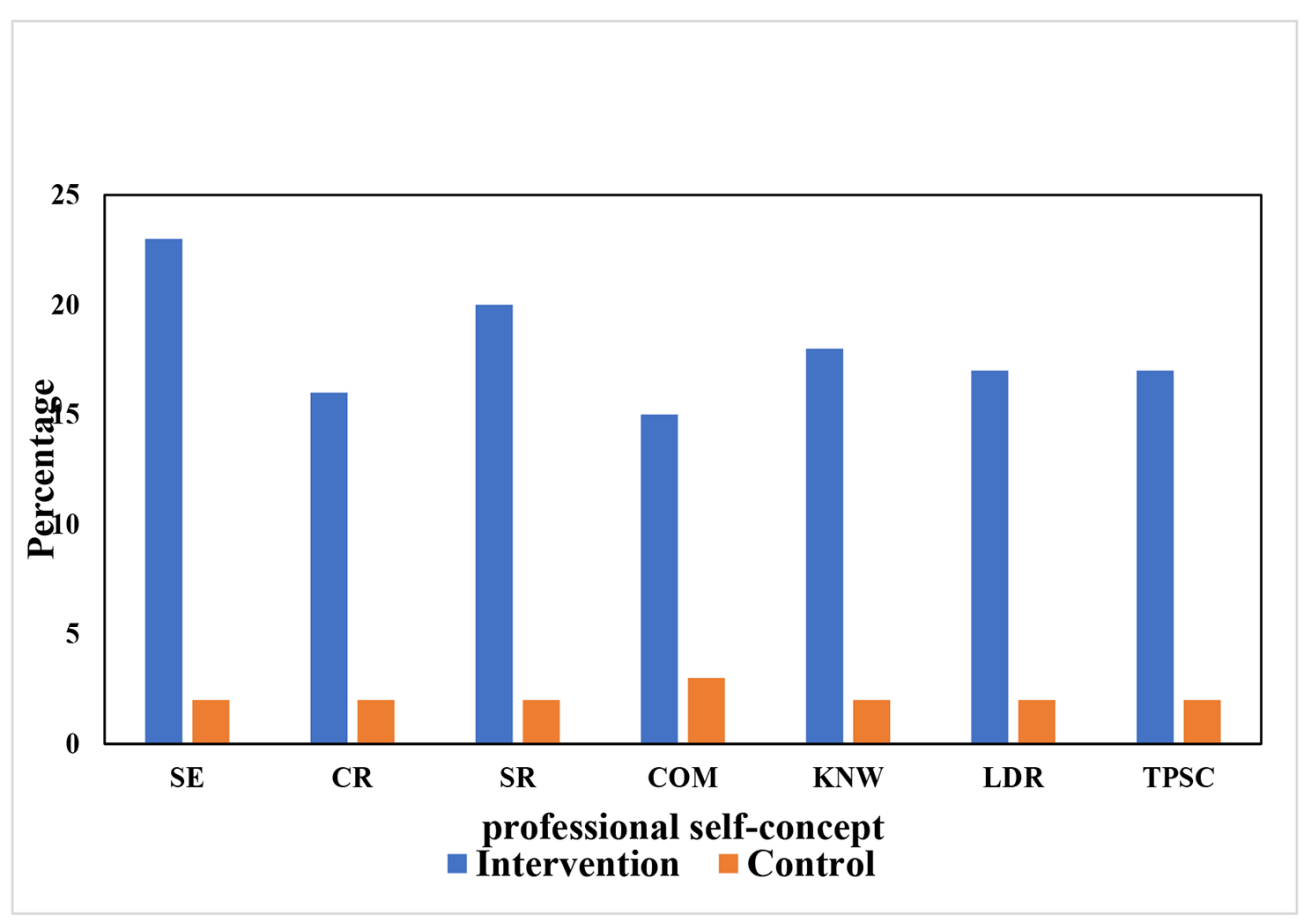
Percentage of improvement in professional self-concept &its dimensions (in control and intervention groups) *SE :Self-esteem CR: caring, .SR: staff relation, COM: communication, .KNW: knowledge, .LDR: leadership, TPSC: total professional self-concept*,

Further, the results of within-group comparison for both control and intervention (Table 4) showed that there were significant differences in PSC and for all its dimensions (self-esteem, caring, staff relation, communication, knowledge, leadership) across the time from pre-test to post-test and follow-up (p < 0.05), and also from post-test to follow-up it was significant (p < 0.05) for both groups. The results of data analysis showed that the percentage of increase in PSC and its dimensions among nursing students under the intervention of the PPL program improved more than in the control group (Fig. 3).

**Table 4:** Pairwise comparison of professional self-concept and its subscales mean score across time

Variable	Group	(I) Test	*(J)* *Test*	Mean Difference (I-J)	SE	P value	95%CI for Difference LB UB		effect size d
Total Professional self-concept	**I**	Pre	Post	-17.28^a^	0.59	< 0.001	-18	-16	6 (Large)
		Pre	Follow-up	-28.27^*^	0.65	< 0.001	-29	-26	
		post	Follow up	-10.98^*^	0.59	< 0.001	-12	-9.8	
	**C**	Pre	Post	-1.87^*^	0.21	< 0.001	-2.2	-1.4	0.2 (Small)
		Pre	Follow-up	-2.77 ^*^	0.27	< 0.001	-3.3	-2.2	
		post	Follow-up	− 0.909^*^	0.23	< 0.001	-1.3	-0.5	
Self-esteem	**I**	Pre	Post	-4.71^*^	0.25	< 0.001	-5.2	-4.2	3 (Large)
		Pre	Follow-up	-5.89^*^	0.28	< 0.001	-6.4	-5.3	
		post	Follow up	-1.18	0.12	< 0.001	-1.4	− 0.92	
	**C**	Pre	Post	− 0.22^*^	0.04	< 0.001	− 0.31	.-12	0.13 (Small)
		Pre	Follow-up	− 0.45^*^	0.06	< 0.001	− 0.57	− 0.33	
		post	Follow up	− 0.23^*^	0.04	< 0.001	− 0.32	− 0.13	
Caring	**I**	Pre	Post	-3.28^*^	0.17	< 0.001	-3.6	-2.9	3 (Large)
		Pre	Follow-up	-4.38	0.16	< 0.001	-4.7	-4	
		post	Follow up	-1.09^*^	0.11	< 0.001	-1.3	− 0.86	
	**C**	Pre	Post	− 0.22^*^	0.06	< 0.001	− 0.34	− 0.09	0.08 (Small)
		Pre	Follow-up	− 0.46^*^	0.08	< 0.001	− 0.63	− 0.3	
		post	Follow up	− 0.24^*^	0.05	< 0.001	-3.6	− 0.1	
Staff relation	**I**	Pre	Post	-3.27	0.20	< 0.001	-3.6	-2.8	2.78 (Large)
		Pre	Follow-up	-5.31 ^*^	0.27	< 0.001	-5.8	-4.7	
		post	Follow up	-2.03 ^*^	0.22	< 0.001	-2.4	-1.5	
	**C**	Pre	Post	− 0.40^*^	0.09.	< 0.001	− 0.59	− 0.21	0.35 Moderate
		Pre	Follow-up	− 0.48^*^	0.09	< 0.001	− 0.67	− 0.28	
		post	Follow up	− 0.07	0.09	0.403	− 0.26	0.104	
Communication	**I**	Pre	Post	-2.22	0.16	< 0.001	-2.5	-1.9	3 (Large)
		Pre	Follow-up	-4.18	0.14	< 0.001	-4.5	-3.8	
		post	Follow up	-1.96	0.13	< 0.001	-2.2	-1.6	
	**C**	Pre	Post	− 0.51^*^	0.06	< 0.001	− 0.65	− 0.38	0.65 Moderate
		Pre	Follow-up	− 0.87 ^*^	0.1	< 0.001	-1.07	− 0.66	
		post	Follow up	− 0.35^*^	0.06	< 0.001	− 0.47	− 0.2	
Knowledge	**I**	Pre	Post	-3.2 ^*^	0.19	< 0.001	-3.6	-2.8	3 (Large)
		Pre	Follow-up	-4.85 ^*^	0.19	< 0.001	-5.2	-4.4	
		Post	Follow up	-1.59 ^*^	0.19	< 0.001	-1.9	-1.2	
	**C**	Pre	Post	− 0.38 ^*^	0.06	< 0.001	− 0.52	− 0.25	0.83 (Large)
		Pre	Follow-up	− 0.67 ^*^	0.10	< 0.001	− 0.88	− 0.47	
		Post	Follow up	− 0.28 ^*^	0.08	0.001	− 0.45	− 0.11	
Leadership	**I**	Pre	Post	-2.98 ^*^	0.21	< 0.001	-3.4	-2.5	2.78 (Large)
		Pre	Follow up	-4.61 ^*^	0.23	< 0.001	-5.07	-4.1	
		Post	Follow up	-1.63 ^*^	0.23	< 0.001	-2.09	-1.1	
	**C**	Pre	Post	− 0.24	0.04	< 0.001	− 0.34	− 0.15	0.81 (Large)
		Pre	Follow up	− 0.44 ^*^	0.07	< 0.001	− 0.58	− 0.29	
		Post	Follow up	− 0.19 ^*^	0.06	0.002	− 0.31	0.06	

* I: Intervention, C:Control

## Discussion

This study was the first to be demonstrated the blended PPL program among undergraduate nursing students in Iran. This study aimed to examine the effect of the PPL program on PSC among undergraduate nursing students during Geriatric-Adult internship clinical practice. The outcomes showed significantly improved PSC development with high effect size and findings imply the effectiveness of the blended PPL program.

### The effect of PPL program on total PSC

The PPL is one method for enhancing nursing professionalism [25]. The results showed a significant increase in total PSC mean scores after intervention and were in line with the finding of some studies in Iran [8, 36] and others countries [37, 38]. The possible explanation for increased total PSC after implementation intervention in the current study is that, this PPL holistic education with blended modality unites active student-centered self-directed learning and guided based on the principles of many theories and models. The blended PPL program facilitated teacher-student face-to-face and online relationships through performing some professional learning modules such as portfolio learning, reflective learning, and case simulation learning practice. Student-centeredness and flexibility of blended PPL in performing reflective practice based on HRM model can help promote critical thinking and critical reflection as requirements for the development of PP during professional nursing practice and lead to development of PSC. [28]. The use of a holistic model combined with integrative and structured reflective activities supports the scaffolder and developmental nature of reflection [25]. Moreover, nursing students’ attitudes regarding reflection influence students’ perceptions of professional self-concept value. Implementation of case study in PPL program based on principles of CPD model provided learners with the chance to analyze the real geriatric and adult patient scenario, use their knowledge in practice, and be actively involved in participatory activities and group discussion. Some studies have been conducted in this field, corroborating the results of the current study. [39] [40] [41]. However, in contrast to the conventional learning method, which could be pragmatic and reductionist and students create little attempt for achieving their learning requirements. Hence, it seems that all the mentioned factors have been involved in strengthening the effectiveness of this method in comparison with the conventional learning method.

### Effect of PPL program on dimensions of PSC

Gaps between theory and clinical practice mean challenges for nursing students through their learning strategies. Professional portfolio learning (PPL) may assist nursing students to improve in knowledge, skills, attitudes, self-learning, study satisfaction, critical thinking, problem-solving skills, and professional competency [19–26]. The results of current study showed the self-esteem dimension before the intervention was the least PSC dimension and after performing the PPL program it was much improved. In addition, the mutual effect of time and group was significant to change in the scores of self- esteem and this effect was more in the intervention group. This result was consistent with other studies [8–11, 42]. The discoveries of this consider appeared that the utilize of a case study model in clinical practice for nursing students improves self-esteem to the learning method, upholds basic audit and reflection, and created the guidance of learning process, and makes it more goal-oriented and orderly [30]. Nursing students with low self-esteem have more problems in communication with peers and patients. They had reduced empathy, efficacy, and low skill performance [19]. Therefore, the lowest mean score for the self-esteem dimension might be since nursing students entering professional clinical practice recognized that clinical settings are strongly physician-centered and may restrict the nurses. Therefore nursing students sense they couldn’t apply their enough capacities and may only implement the physicians’ orders, and could not do nursing intervention independently. As a hidden curriculum, this culture maybe leads to low self-esteem in nursing students. After the implementation of PPL, students performed a clinical simulation seminar and reflective practice regarding their geriatric adult patients that help to adapt to the stressful clinical environment and improve self-esteem dimensions. This can also assist in significant learning from their peers and so reduce the theory-practice gap.

However, the leadership dimension was least improved compared with the rest of the PSC dimension, but it was a significant difference. This result was consistent with results of recent studies indicated students who participated in purposeful workshops and clinical activities such as the reflective practice and simulation scenarios [43] improved the students’ teamwork skills attitudes [44] and the use of clinical scenarios facilitates effective education and advances students’ problem-solving, critical reasoning, and analysis abilities [45]. In this way, performing these blended professional learning modules through the development of PP in the current study meet the desires of undergraduate nursing students to the development of PSC dimensions such as self-esteem, knowledge, and caring and plan them for clinical practice, representing a chance to fulfill these gaps [9]. In addition, during the program, nursing students not only it was communicated with their facilitator and researcher but also participated in group discussions together with their peers and exchanged information with each other. Peer learning or learning together may be a critical factor behind the advancement of learning and critical thinking [46]. Hence, the blended PPL program with different professional activists could improve PSC components such as communication, staff relation, and leadership in the intervention group.

In contrast, results presented no important growth in the mean scores of total PSC and its dimensions in the control group. The conventional learning strategy was seemed not to simulate the Iranian undergraduate nursing students to expand their PSC during their professional practice and thereby learners make a little attempt for fulfilling their educational needs. The discrepancy could be due to the dissimilar ways of bringing the learning program. There are many criticisms of conventional learning. Through conventional learning, instructors give students an enormous sum of information in a brief sum of time without the capability to proficiently interface with them, hence, they ordinarily confront challenges .particularly in caring of geriatric patients with different health issues, in making a strong clinical environment for the development of PSC. However, the control had experienced a slight increase in the total mean score of PSC. The possible explanation may be changes in the work environment situation, communication between the students, peers, staff at work, or the inspiration and activity of the control group through using a logbook. Both learning strategies offered and allowed the students to practice PSC as they were exposed to the related conditions in the clinical setting. Nonetheless, the effect of each education strategy was different. Therefore, incorporating a blended teaching-learning approach in this study was a very effective approach and so the outcomes attained were in line with those found in the researches of some studies by [16, 22]. But interaction might not be sufficient in other PL studies as compared to the blended intervention.

### Strengths and limitations of study

One of the strengths of the study was using the PPL as one approach for enhancing nursing professionalism [25] and monitoring professional development through the guidance of theoretical framework and models such as implementing the professional development activities CPD model, reflective practice, and a long intervention of sixteen weeks. Since PPL was created over time, it offered a way of monitoring professional development. It should be noted that the portfolio was delivered in sixteen weeks, allowing the nursing students to become more improve their professional self-concept. Another strength was blended learning of the sixteen weeks’ face-to-face and online intervention may have allowed them to build skills incrementally with feedback weekly. Students can access information in the online Telegram platform at any time, so led to motivation and personalization which is not possible for control group interaction.

The current study was limited to fourth-year nursing students only. Since the self-report questionnaires do not describe the real situation of participants so may present bias to study outcomes, they might rate higher or lower than the real condition. However, using the self-reported questionnaire in the current study provides a basis for professional self-concept among the Iranian undergraduate nursing students and there is no information about them previously. Another limitation of the study is the participants in the groups are fully aware of their participation in the current study and this may have introduced bias to results, but the researcher reminded them to maintain behavior naturally. Last, the current study was a quasi-experimental study with quantitative research methods, further research with qualitative or mixed research methods can be carried out to examine the effects of PPL with blended learning in more depth. Besides research employing a qualitative study can be utilized to investigate in detail the most excellent conveyance strategy for the PPL program, and the components that influence the nursing students’ learning process with the intervention.

## Conclusion

This professional portfolio learning program demonstrates as an innovative and holistic blended teaching-learning approach to improve professional self-concept and its dimensions during professional clinical practice among undergraduate nursing students. The blended teaching-learning approach of delivering the PPL (face to face and online) during the workshops and clinical practice is a practical, cost-effective, and less time-consuming way to train professional development activities of PPL to nursing students with busy schedules during internship professional clinical practice. It appears that the use of a blended designed of professional portfolio can promote a link between theory and the advancement of geriatric adult nursing internship practice. The data obtained from the present study can be useful for nursing education to evaluate and redesign a curriculum for development of nursing professionalism as a quality improvement process and groundwork to develop new models of teaching-learning and assessment.

## Data Availability

The datasets generated in the current study are available from the corresponding author upon reasonable request.

## List of Abbreviations

PSC: Professional Self-Concept
PP: Professional Portfolio
PPL: Professional portfolio learning
NPSC: Nurse Professional Self-concept
HRM: Holistic Reflection Model
CPD: Continuing Professional Development
PLG: Portfolio Learning Group
GEE: Generalized Estimating Equations
GPA: Grade Point Average
BSN: Bachelor of Sciences in Nursing
